# The impact of climate variability on agricultural employment in Mexico from 1980–2017

**DOI:** 10.1371/journal.pone.0313891

**Published:** 2025-02-10

**Authors:** Karla Arlae Sánchez Guijosa, Guillermo Murray-Tortarolo, Mario Martínez Salgado

**Affiliations:** 1 Instituto de Investigaciones en Ecosistemas y Sustentabilidad, Universidad Nacional Autónoma de México (UNAM), Morelia, Michoacán, Mexico; 2 Posgrado en Ciencias de la Sostenibilidad, Universidad Nacional Autónoma de México (UNAM), Morelia, Michoacán, Mexico; 3 Unidad de Investigación sobre Representaciones Culturales y Sociales, Universidad Nacional Autónoma de México (UNAM), Morelia, Michoacán, Mexico; Xinjiang Institute of Ecology and Geography (XIEG), CHINA

## Abstract

Employment in the agricultural sector is highly dependent on climate. Most agricultural jobs worldwide rely on predictable precipitation, in terms of both quantity and seasonality. Mexico is a largely agrarian country, with at least 20 million people directly reliant on food production for the livelihoods. However, research on the relationship between climate variability and agrarian employment is limited in the nation, complicating the development of effective adaptation strategies to drought and climate change. This study aims to address this gap, by analyzing the employment changes of farmers and livestock producers at a national level in the past five decades (1980 to 2017) and its relationship to long-term precipitation variability. We employed governmental datasets from national agrarian surveys and national precipitation, both at the annual scale and seasonally within each year. We found a negative relationship between agricultural employment and total annual precipitation. In particular, employment in the livestock sector showed a negative correlation with current-year precipitation (p = 0.06, cor = -0.33), while employment in rainfed agriculture was linked to the previous year’s rainfall (p = 0.07, cor = -0.33). It is likely that this pattern was driven by the positive relationship of precipitation with planted cropland area (p<0.05, cor = 0.19) and agrarian income (p<0.05, cor = 0.18). We also found that as many as 10 million people left the agrarian employments each year during the dry season. Finally, as precipitation continues to pose a challenge, it may have contributed to people of ages 23 to 35 to leave in recent years, compared to 15 and 19 in the 1990s. These findings underscore the need for national policies to mitigate the impacts of dry years on livelihoods and to inform strategies for building resilience in the agricultural sector.

## 1. Introduction

Modern agriculture and the livelihoods of rural farmers remain highly dependent on climate. In recent years, studies have attributed the changes in crop yield variability to climate change [1]. For example, Sultan et al. [2] demonstrated the negative effects of global warming on millet and sorghum crop yields in West Africa, and Moura da Silva et al. [3] documented the yield risks of soybean cultivation in Brazil. The impact of extreme events are not limited to developing nations, but have also been widespread in developed regions such as Europe [4, 5], North America [6, 7], and Australia [8, 9]. This situation threatens the progress of global food security, as well as the livelihoods of people who depend directly on this activity [10]. Climate change, therefore, jeopardizes not only food production but also social structures.

There are several examples of the cascading effects of climate variability in modern agriculture, and the livelihoods of people who depend on it. One such example is the ongoing conflict in Syria that began in 2011. The intensification of this conflict has been correlated with one of the worst droughts the country has faced. It is argued that the drought exacerbated the already existing water and agricultural insecurity, as it caused massive agricultural failures and increased livestock mortality [11]. Another example is sub-Saharan Africa; where it has been observed that dry precipitation anomalies reduce the number of violent events between armed actors; however, extremely dry conditions increase riots and violence against civilians [12]. Interestingly, these types of conflicts may result from an immediate response of the population to meteorological stress [12]. One last example is the impact of precipitation on children’s education. Higher than normal rainfall has been found to correlate negatively with the educational attainment of children in Central America and the Caribbean; likely because children experience poorer health and nutrition as a result of flooding and crop failure [13]. Thus, climate variability is still a key demographic driver for most modern societies.

At the core of this link between climate variability and demographic patterns, is employment in the agricultural sector [14]. Globally, most agricultural jobs depend on predictable rainfall, both terms of quantity and seasonality [15]; thus, significant deviations from regular climatic patterns can lead to the abandonment of these activities [16]. Several examples of this phenomenon exist in different parts of our planet. In Nigeria, reduced rainfall initially raises agricultural employment, as farming requires more labor in dry conditions; however, protracted droughts significantly reduce agricultural jobs due to lower crop yields [17]. In the United States it has been shown that drier than usual weather conditions negatively impact the total wages paid to agricultural employment on crop-producing farms, and reduces net occupation in the primary sector [18]. A final example is Mexico, where it has been shown that storms can have significant negative associations with labor market outcomes because of reduced agricultural productivity [19]. Thus, there is theoretical evidence exposing the impact of climate extremes on employment.

Mexico has been identified as one of the most vulnerable countries to climate change, particularly in terms of its agrarian structure [20]. During extreme dry years the country has experienced decreases in rainfed agriculture production [21] and cattle populations [22]. An example of this was the 2011–2012 drought years in which cattle and goat populations declined by about 3% [23], and maize production decreased by 17%. In other words, Mexican agriculture is highly dependent on rainfall; years with good rainfall translate into high agricultural production (both crops and livestock products), and vice versa. The impacts of severe weather in Mexico are not limited to agricultural production but also have a profound effect on the livelihoods of farmers. Recent studies have reported that migration from Mexico to the U.S. increased as a consequence of decreased precipitation [24]. Other studies have observed that in a drought year, the probability of migrating to the U.S. increases [25]. The impact of dry conditions is not uniform across Mexico; droughts have a greater influence on emigration from rural areas in states with dry climates [26].

One of the most important demographic processes in Mexico is the change in agrarian employment. Throughout history, the primary sector has been considered a significant component of the country’s economy, history, and politics; however, its importance is declining [27, 28]. Some researchers attribute this decline to the country’s development process, where a constant reduction has been observed [29]. This is partially the result of a large portion of the agricultural labor force shifting to the manufacturing or service sectors [27, 30]. However, determining the causes of changes in agricultural employment is difficult, as the challenges faced by farmers and livestock farmers are not solely due to socio-political and economic factors that limit their market inclusion [31, 32]. The shift in occupation is also likely influenced by climate. For example, it has been observed that during the period between 1980 and 2007there was a reduction in local employment by up to 1.4% linked to high heat incidence [33]. However, there is no long-term, nationwide longitudinal research that has studied the impact of climate variability on agrarian occupation.

The objective of this project is to analyze how climate variability correlates with occupational changes in the Mexican agricultural sector over the last four decades (1980–2017). We studied this over an extended period and at the national scale, comparing occupational changes (per year and per quarter) with annual precipitation. We were particularly interested in identifying how drought altered occupations in key vulnerable groups, such as farmers and livestock farmers, considering the total population surveyed.

## 2. Methodology

### 2.1 Study area

#### 2.1.1 Biophysical characteristics

Mexico, situated in the southwest of the North American continent [34], is a mountainous country spanning just under two million square kilometers [35]. Approximately two-thirds of its land area experiences arid or semi-arid climates, while the remaining third is characterized by humid climates [36]. Climate variability is strongly influenced by factors associated with the topography of the terrain and sea surface temperature [37]. For example, precipitation depends, among other factors, on phenomena such as El Niño and the hurricane season in the North Atlantic and Pacific [38]. Researchers have determined that Mexico’s geophysical features contribute to its highly complex and heterogeneous landscapes [39]. These features facilitate the convergence of two biogeographic zones, the Nearctic and the Neotropical, fostering the development of species with temperate and tropical affinities [40]. Consequently, the soils exhibit high fertility levels, supporting various economic activities such as agriculture [41].

#### 2.1.2 Socioeconomic characteristics

To comprehend Mexico’s vulnerability to climate change, it is essential not only to analyze its geographical location but also to examine the high levels of poverty and the skewed income distribution within the country [35]. Poverty is not merely the absence of income; it represents the inability of individuals to fully participate in society and the deprivation of the freedom to satisfy hunger, receive education, and maintain good health [42, 43]. Income distribution, on the other hand, involves providing equal opportunities for individuals to attain desired capabilities [44]. In Mexico, between 2008 and 2018, the percentage of the population living in poverty at the national level decreased from 44.4% to 41.9% [45]. In other words, in 2018, approximately 42 out of every 100 people experienced at least one social deprivation and had insufficient monthly income to acquire necessary food, goods, and services. By 2022, this figure had reduced to 36 out of every 100 individuals [46]. Moreover, it has been reported that since 2014, two-thirds of the wealth has been concentrated in the hands of the top 10% of the country’s richest, with the top 1% of the very wealthy holding more than a third [44]. It is worth noting that the impact of poverty and income distribution is not uniform; there is a higher likelihood of accessing resources if one comes from the rural sector [46]. This context is particularly pertinent considering that approximately 26 million people in rural regions depend directly or indirectly on agrarian occupations [20, 47].

#### 2.1.3 Employment definition

Employment, as defined by INEGI [48], refers to any situation in which individuals, regardless of their income level, engage in activities under the assumption that there is demand for their goods or services. This includes agricultural employment, which covers both seasonal workers hired to prepare the land and those involved in year-round food production through crops or livestock.

### 2.2 Datasets

#### 2.2.1 Precipitation

To identify climate variability in Mexico, annual precipitation data at the national level were obtained from the Climate Research Unit [49]. The technique used for data analysis involved calculating the average annual and quarterly precipitation for the entire period, as well as the annual anomalies, defined as the differences between the precipitation of each year and the average of the entire period (1980–2017).

#### 2.2.2 Employment

We used employment data from three databases generated by the National Institute of Statistics and Geography (INEGI): the Demographic Retrospective Survey (EDER), the National Occupation and Employment Survey (ENOE), and the National Employment Survey (ENE).

The EDER is a longitudinal study created by INEGI and other academic institutions. The purpose of this survey is to collect longitudinal data that summarizes the life history of individuals, focusing on various sociodemographic processes such as migration, education, employment, fertility, mortality and more [50]. There are three EDER surveys; conducted in 1998, 2011 and 2017 [51]. In this research, we used the EDER 2017, which, unlike the previous surveys, provides representative information at the national, urban, and rural levels [51], encompassing 32,000 household interviews. The methodological strategy of EDER 2017 consists of recording various events that occurred at least once per year in their lifespan, placing them both in historical time and in lifetime [51]. In other words, the survey reconstructs a person’s life based on the information provided, aligning it with the individual’s age and the calendar years. It is worth mentioning that the information available is about the life history of women and men born between 1962 and 1997.

The ENOE is a quarterly survey aimed at obtaining statistical information on the occupational characteristics of the population aged 15 years and older at a national scale, as well as demographic and economic variables for labor force analysis [52]. A key feature of the ENOE is its rotating panel structure, where each individual is surveyed five times before being replaced [53]. In other words, the ENOE is a continuous survey designed to yield quarterly results at the national, state, and city levels, including 36 self-represented cities [53]. It includes two forms: the Sociodemographic Questionnaire and the Occupation and Employment Questionnaire [52]. The first form serves to identify household members, recording their sex, age, education level, and other basic details, irrespective of their engagement in economic activities [52]. The second form categorizes the working-age population into two groups: the Economically Active Population and the Non-Economically Active Population [52]. The total sampled population was 150,000 households per quarter.

The ENE was conducted in Mexico from 1988 to 2004 and is the precursor of the ENOE. The survey aimed to establish a statistical information base on national-level occupational characteristics of the population, along with other demographic and economic variables, to facilitate in-depth labor analysis [54]. A notable feature of this survey is its temporal evolution: initially conducted biannually, then annually, and, starting in 2000, continuously with quarterly results publication, leading to the development of the ENOE [55]. Another characteristic shared by the ENE and the ENOE is the rotation of the sample in each survey. In other words, both surveys periodically replace a portion of the sample with new participants of similar characteristics to ensure reliable labor force, occupation, and employment estimates. Consequently, the total sampled population fluctuated between 1988 and 2005, ranging from 46,613 households in 1988 to 163,838 in 2000 [55]. This dataset was used solely to compute changes in total agrarian employment; we did not correlate these results with climate data, as the ENOE dataset was used for that purpose.

**Fig 1:**
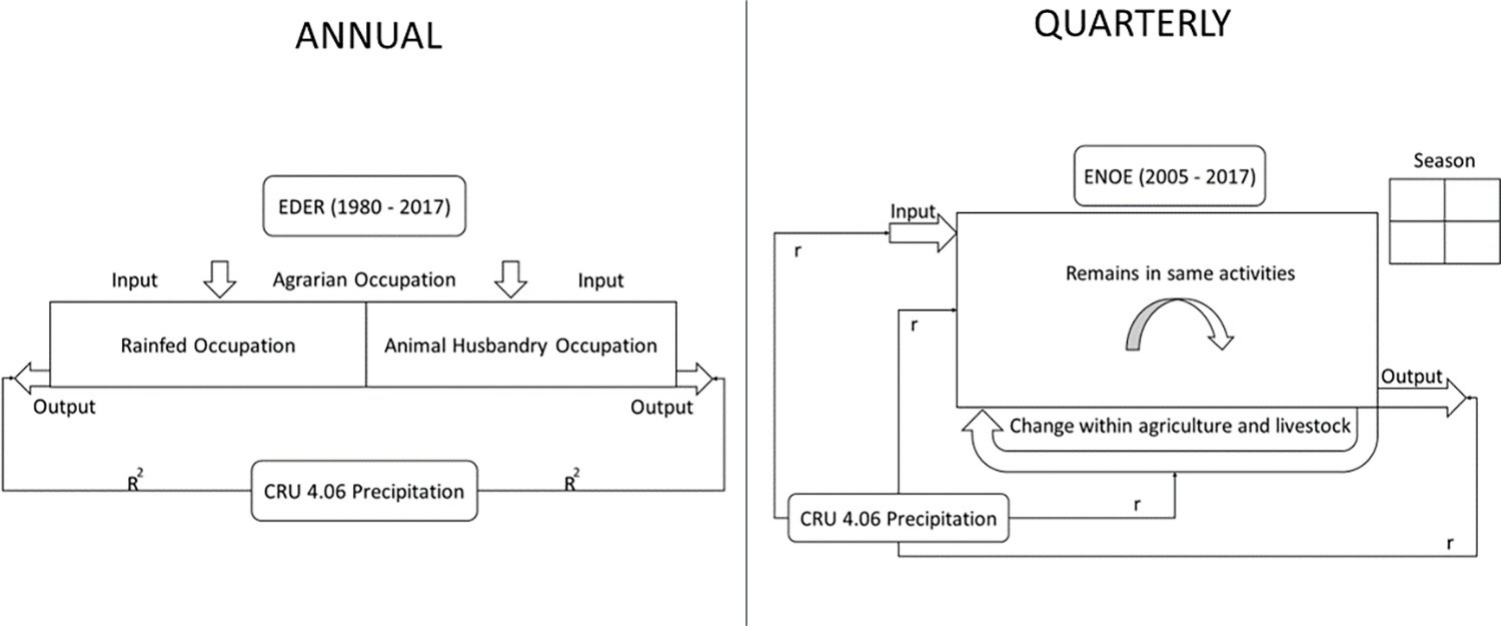
Methodology. Depicts a national-scale system under investigation. Precipitation analysis was conducted using the CRU 4.06 database, while two different databases, EDER and ENOE, were employed to analyze occupational changes. The survey’s temporal and methodological aspects enable population monitoring on an annual and quarterly basis, allowing for two distinct scales of comparison. Specifically, for EDER 2017, we compared the annual data of total agrarian occupation, rainfed occupation, and livestock occupation with the average annual precipitation (left). In the case of ENOE, we utilized quarterly average data for occupation and precipitation transitions (right). In both analyses, a simple Pearson correlation was employed to compare variables and determine statistical significance.

In summary, we used three databases to calculate changes in overall agrarian occupation. However, to analyze the correlation between precipitation and changes in occupation, we employed only the Demographic Retrospective Survey database (EDER, 2017) [50] and the National Occupation and Employment Survey databases (ENOE, 2005 to 2017) [56]. This decision was based on the sample design: EDER allows for the reconstruction of the population’s long-term life history, despite having less data. In contrast, ENOE provides a more detailed perspective on short-term changes, facilitating the analysis of annual movements. While its sample size is significant, it does not support individual tracking beyond five quarters. In other words, the analysis of these databases complements one another, as they enable the visualization of occupational changes on an annual and quarterly basis (Fig 1).

*2*.*2*.*2*.*1 Ethics statement*. Considering that our research includes the participation of human beings, we clarify that we did not obtain consent, either written or oral, as fieldwork was not conducted. The data used were sourced from publicly available information obtained from the National Institute of Statistics and Geography (INEGI). In accordance with Article 26.B of the Political Constitution of the United Mexican States (CPEUM), INEGI is an autonomous agency with legal personality and assets, empowered to regulate the collection, processing, and publication of information, ensuring compliance [57].

Aligned with the principles outlined in the CPEUM, particularly Article 6, Base A, Sections I and II, INEGI upholds maximum transparency in disseminating information [57]. However, personal data is safeguarded in accordance with the provisions and exceptions stipulated by law. Pursuant to Articles 33 and 35 of the General Law for the Protection of Personal Data in Possession of Obligated Parties (Data Protection Law), INEGI is committed to upholding individuals’ rights to data protection, ensuring compliance with legal principles such as legality, purpose, loyalty, consent, quality, proportionality, information, and responsibility [58].

As an Obligated Entity, INEGI assumes responsibility for the treatment and protection of personal data under Article 1 of the Data Protection Law [58]. It is mandated to establish and maintain administrative, physical, and technical security measures to safeguard personal data against unauthorized access, use, alteration, destruction, or loss, ensuring confidentiality, integrity, and availability in accordance with Article 31 of the aforementioned law [58].

### 2.3 Data analysis

#### 2.3.1 Change of employment

The two databases (EDER and ENOE) have different temporalities and methodologies for data collection; therefore, two different methods were used to analyze changes in employment. In EDER 2017, we selected six variables to facilitate information handling. We were interested in the ID of each person interviewed, their age, their sex, the retrospective year and the categories for annual and temporal occupations. From this shortened dataset, we selected the records of persons who were 12 years of age or older in any given year and reclassified the individuals into three occupation categories: unemployed (either unemployed or housemakers), general workers, and those who worked in the agricultural sector. This was done for each living year of each interviewed individual.

Regarding the classification of occupation categories, it is important to note that these classifications have already been assigned by the National Institute of Statistics and Geography (INEGI). For the analysis of the EDER, the National Occupational Classification System (SINCO) [59] was employed. Specifically, the group of "Workers in Agricultural and Livestock Activities" was scrutinized, with a particular focus on rainfed agriculture and grazing livestock activities. Therefore, our classification encompasses farmers (engaged in rainfed occupations), livestock farmers (involved in animal husbandry occupations), and agrarian occupations (considering both rainfed and animal husbandry activities).

We identified all possible changes within these categories, including workers who changed occupations within agriculture. This means that we analyzed the activities each respondent undertook in one year and checked for any changes in the following year. For instance, respondents might have shifted from cultivating crops to engaging in livestock activities. Respondents were only classified as having left the agricultural sector when their activities were no longer associated with farming. Subsequently, we calculated the rate of change by comparing the number of individuals who changed their occupation within agriculture to the total number of respondents employed in the sector. The EDER 2017 sample consists of an average of 8,434 employed persons per year. Of these, an average of 1,198 persons per year were engaged in the agricultural sector. Within this group, the surveyed population focusing on agriculture numbered 1,068 annually on average. Meanwhile, the average number of livestock farmers interviewed per year was 130. Despite this being the largest demographic survey available at the national level, data are sparse for years before 1990, particularly for livestock farmers, and should be interpreted carefully. We decided to include all decades in the analysis as we are looking at nationwide processes, but the intrinsic limitations of the survey make it impossible to scale down to regions or states.

For the ENOE (2005 to 2017) [56], data are presented quarterly. Therefore, we identified the main occupation of each respondent for each period within the year, focusing on those who worked in agricultural activities for at least one quarter. Thus, the ENOE sample (2005–2017) comprises an average of 583,132 employed persons per year, of which 55,134 are engaged in the agricultural sector. After obtaining the occupations by quarter, we compiled five possible transitions for each period: 1) No change: people who remained in the same activity outside agriculture, 2) Output: individuals who left agricultural activity, 3) Input: those who started working in agriculture, 4) Remained in the same agricultural activity, and 5) Those who changed activities within agriculture (e.g. from agriculture to raising cattle; or from maize to alfalfa; or from food crops to cash crops / plantations).

Regarding the classification of occupation categories, it is important to note that two classifiers developed by INEGI were utilized. Specifically, from 2005 to the second quarter of 2012, the Mexican Classification of Occupations (CMO) [60] classifications was employed. However, starting from the third quarter of 2012, there was a transition to the National Occupational Classification System (SINCO) [59], which provided a higher level of description for various activities. This shift allowed for more detailed distinctions, such as the inclusion of "Support Workers in Agricultural Activities" as a recognized category.

For the ENE (1998 to 2004) [54], the data are presented both annual and quarterly, so we chose to use the interactive tabulations provided by INEGI for the National Employment Survey. We selected the employed population tabulation and then applied the filters “Main Occupation” and “Total Workers” to identify the number of respondents employed in an agricultural activity.

#### 2.3.2 Statistical analyses

*2*.*3*.*2*.*1 Agrarian abandonment*. Drawing from the shortened dataset of EDER 2017, which exclusively includes individuals aged 12 and above, along with the reclassification of unemployed individuals, general workers, and those engaged in the agricultural sector, we conducted an analysis to ascertain the average age of departure from agricultural occupations. Our aim was to rule out the possibility that abandonment is related to age. To accomplish this, we documented instances of individuals transitioning out of farming over their lifetimes and visualized this data using a time series graph.

Using the same abbreviated dataset from EDER 2017, we calculated the number of unemployed individuals for each year. This was done to analyze the movements of the Mexican population who left agricultural occupations, particularly to determine whether leaving these occupations resulted in unemployment or a transition to another occupation, either within or outside of agriculture. We counted the number of times individuals exited agriculture over their lifetimes, as well as the number of unemployed individuals during the period under analysis.

To explore how climate variability impacts agricultural employment in Mexico, the relationship between mean annual precipitation (mm) and both the planted area (ha) and the production value of rainfed agriculture (in thousands of pesos) was examined. Precipitation data were sourced from the Climate Research Unit [49], while data on planted area and production values were obtained from the Statistical Yearbook of Agricultural Production (SIAP) [61]. The data analysis involved adjusting the production value to account for inflation, using the index provided by the National Institute of Statistics and Geography (INEGI) [62]. Subsequently, the annual changes in income and planted area were calculated, expressed as percentage variations. Finally, a correlation between mean annual precipitation and changes in the planted area was conducted.

*2*.*3*.*2*.*2 Correlation between climate variability and agrarian occupation*. Two scales of comparison were carried out. First, for EDER 2017, we compared annual data on total agrarian occupation, rainfed occupation and animal husbandry occupation against the mean annual precipitation. We used Pearson’s correlation coefficient to compare these variables and calculated the statistical significance using this method. For ENOE, we employed mean quarterly data on occupation transitions and precipitation, which we correlated using the Pearson coefficient, and also computed the statistical significance with this method. Only significant correlations are presented (Fig 1).

## 3. Results

### 3.1 Long term precipitation and agrarian occupation variability

**Fig 2:**
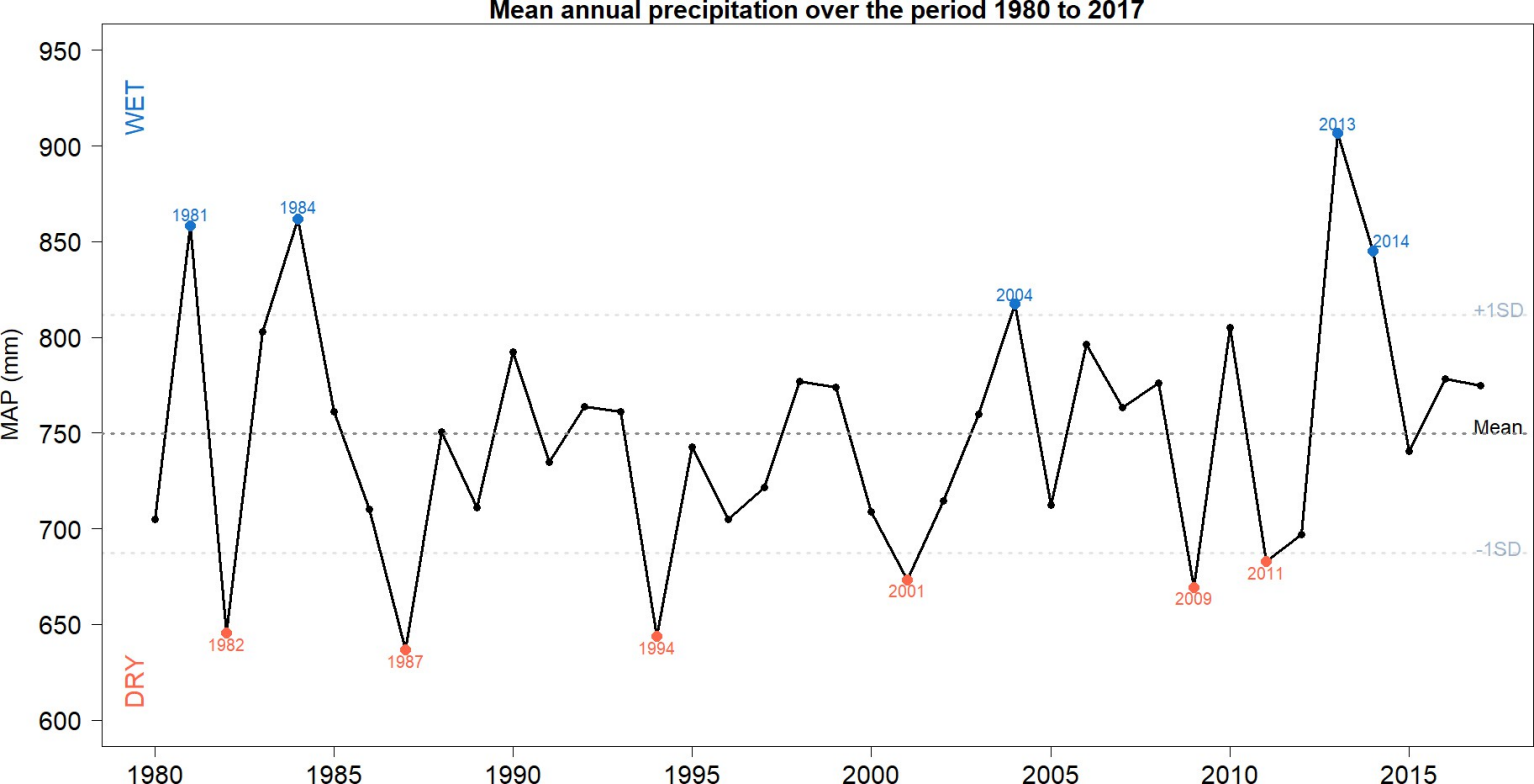
Mean annual precipitation from 1980 to 2017. Years with precipitation above one standard deviation are represented in blue, while years with precipitation below one standard deviation are shown in orange. The wettest year of the entire period is 2013, with 908 mm, and the driest year is 1987, with 636 mm. The dashed line in the center represents the average annual precipitation, and the two dashed lines indicate plus and minus one standard deviation. (Source of data: Calculations by the authors using CRU 4.06 precipitation [50]).

Precipitation in Mexico during the study period displays high interannual variability (Fig 2). The mean annual precipitation during this period was 750 mm, with a standard deviation of 63 mm. In this context, years with less than 687 mm of rainfall can be considered dry (one standard deviation below the average), while years with precipitation greater than 813 mm can be considered a wet year (one standard deviation above).

**Fig 3:**
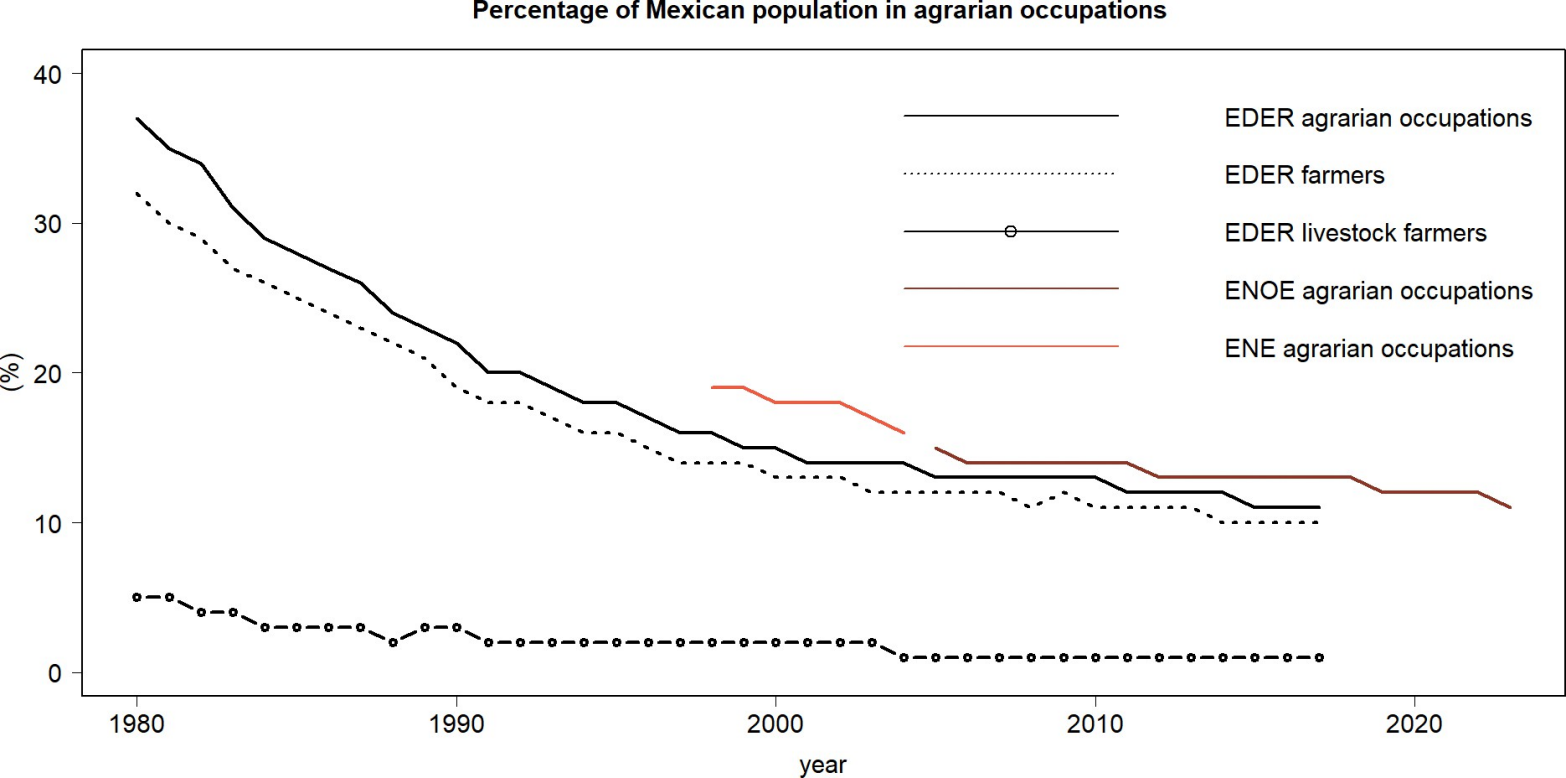
Population in agrarian occupations from 1980 to 2017. The solid black line represents the total agrarian occupation, including all respondents employed in agriculture and livestock from 1980 to 2017. The dashed line represents farmers, while the dotted line represents livestock farmers. The red line shows the agricultural sector according to ENE data for the period 1998 to 2017, and the brown line covers the period from 2005 to 2017 with ENOE data. The figure illustrates a steep reduction in the number of occupations in Mexican agriculture. (Source of data: Calculations by the authors using EDER 2017 [50], ENOE 2005–2017 [56], and ENE 1998–2004 [54]).

When agrarian occupation was analyzed in the long term (1980–2017), we found a continuous decrease in the number of people engaged in such activities (Fig 3). The total number of people employed in an agrarian occupation according to EDER decreased from 37% in 1980 to 11% in 2017. During the same period, rainfed agriculture dropped from 32% to 10%, while livestock farming fell from 5% to 1% respectively. Interestingly, within the population employed in agrarian occupations, 90% are enrolled in agriculture and the remaining 10% in livestock raising across the whole period. This decline is also evident in ENE and ENOE, with the proportion of agrarian occupations decreasing from 19% in 1998 to 16% in 2004, and further to 13% in 2017.

Despite the sharp decline, there is some small interannual variability in the change of occupation. For example, the variation for the entire agrarian occupation sector ranges from -2.2% (in 1980) to +0.1% (in 2008). For agriculture specifically, the variation ranges from -2.1% (in 1980 and 1982) to +0.1 (in 2016); while for livestock, in ranges from -0.6% (in 1983) to 0 in different years. Thus, the sharpest decline is observed in the first decades of the study period, while some stability seems to have appeared in the last two decades. We also observed that the average age of departure from agrarian occupations shows a consistent increment. For example, between 1980 and 1990, it ranged between 15 and 19 years, whereas from 2000 to 2017, it fluctuated between 23 and 35 years. This trend suggests a gradual abandonment of agrarian occupations, where younger individuals left first and are recently followed by older individuals (S1 Fig).

#### 3.1.1 Seasonal variability in agrarian occupation

**Fig 4:**
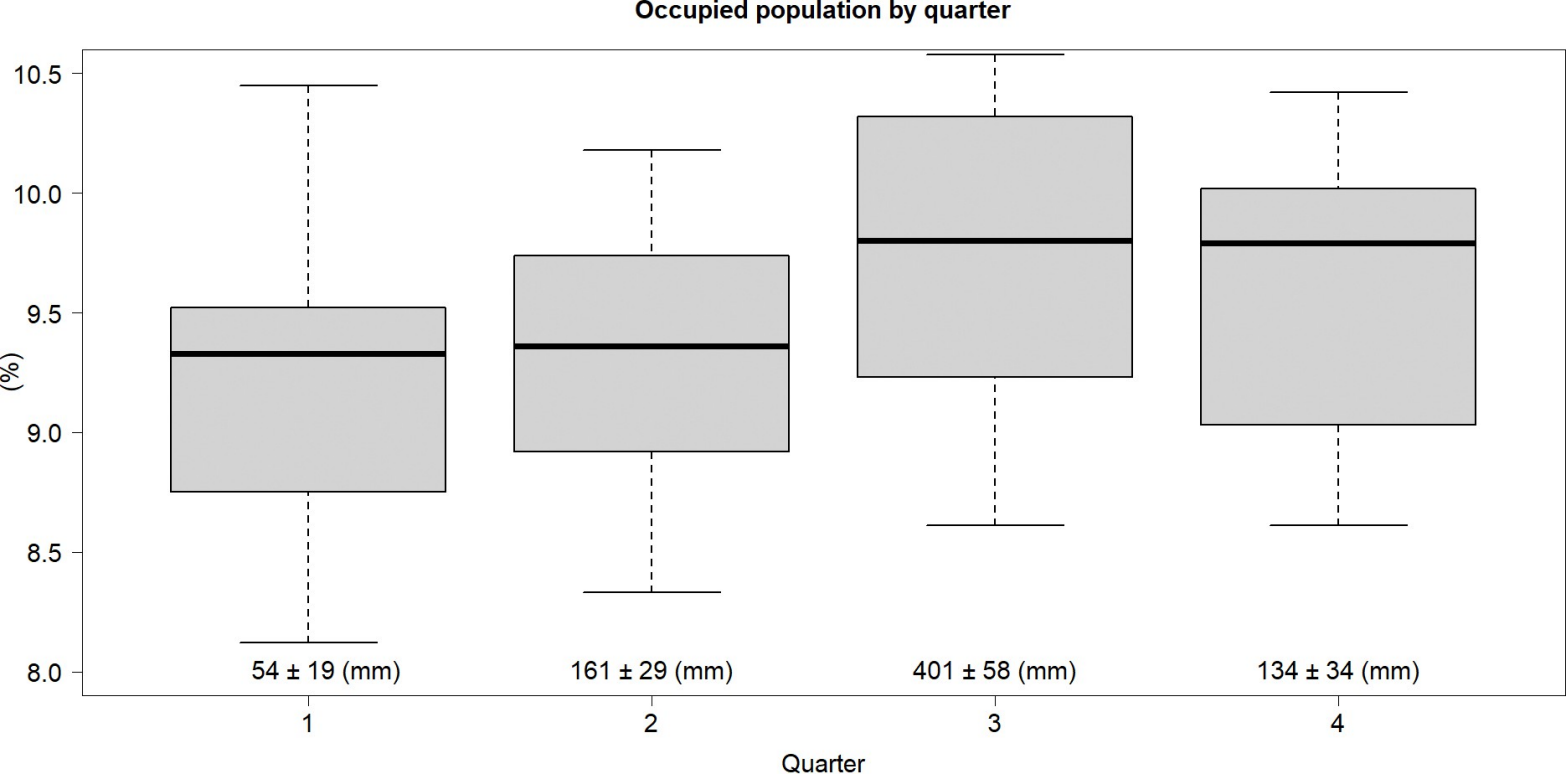
Occupied population quarter. Seasonal variability of the employed population in agrarian occupations was examined by quarter from 2005 to 2017. The third quarter, which aligns with the peak of the rainy season, recorded the highest employment rate at 9.8%. In contrast, the first quarter, coinciding with the peak of the dry season, exhibited the lowest employment rate at 9%. Although the difference of 0.8% may seem small, it translates to approximately 10.5 million people. The precipitation and standard deviation for each quarter are shown below the whisker boxes. (Source of data: Calculations by the authors using ENOE 2005–2017 [56]).

We observed large shifts in agrarian occupation in Mexico within the year. On average over the last13 years (2005–2017), the third quarter (peak rain season) of the year (July-September) had the highest percentage of the employed population in agriculture, with a median of 9.8%, while the first quarter (peak dry season) had the lowest values, with a median of 9% (Fig 4). The second quarter shows a slightly higher value than the first, reflecting an increase in agrarian occupation as the rainy season begins. Conversely, the inverse pattern can be observed in the last quarter, where a decrease in agrarian occupation is correlated with the onset of the dry season. Although the percentage difference between quarters may seem small (0.8%), it corresponds to the seasonal occupation change of approximately 10.5 million people each year.

#### 3.1.2 Interannual variability

**Fig 5:**
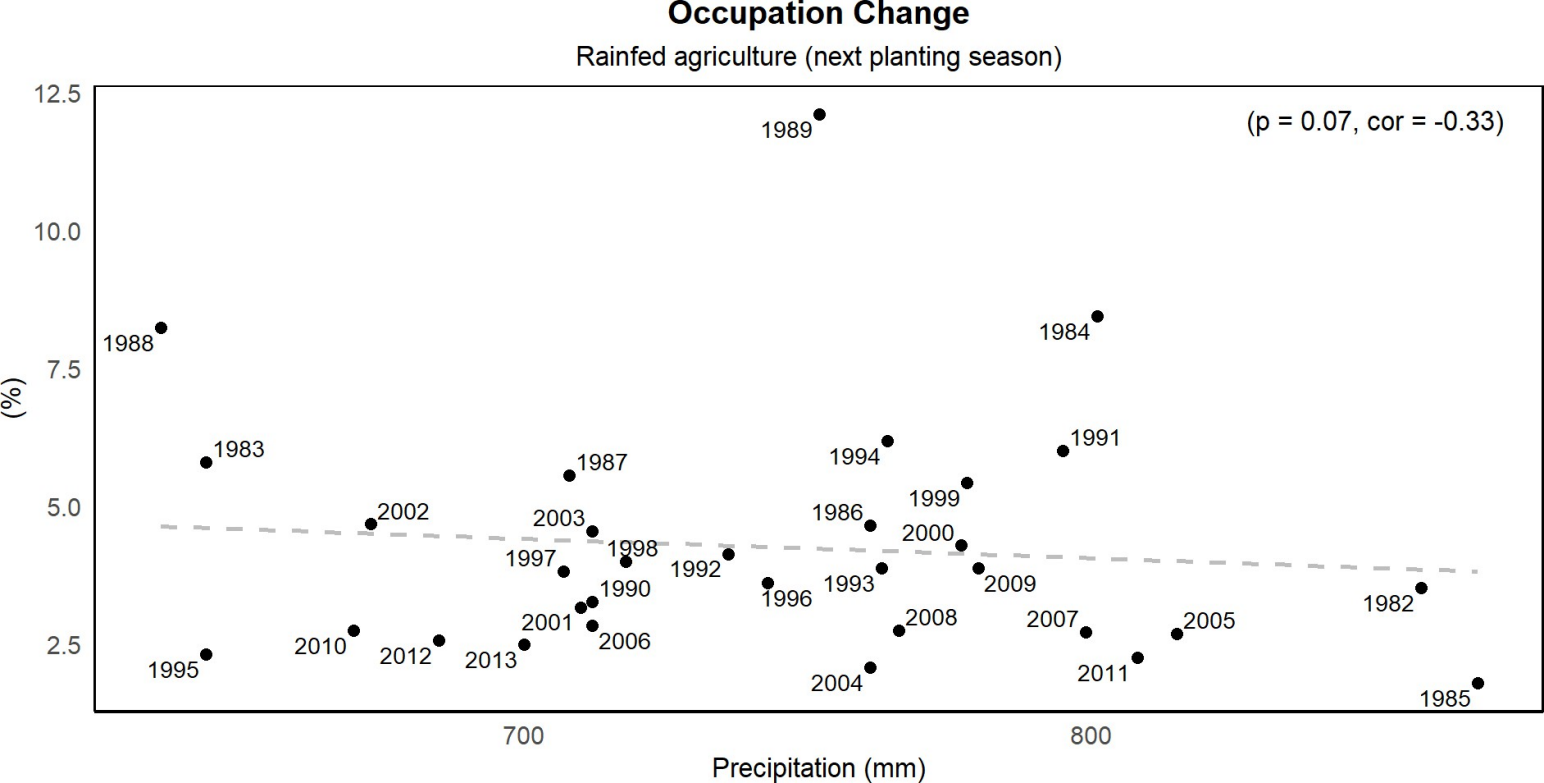
Rainfed agriculture next planting season. Correlation between mean precipitation and rainfed agriculture occupation during the next planting season. The Pearson correlation coefficient and significance test are presented in the top left part of the figure. (Source of data: Calculations by the authors using EDER 2017 [50]).

**Fig 6:**
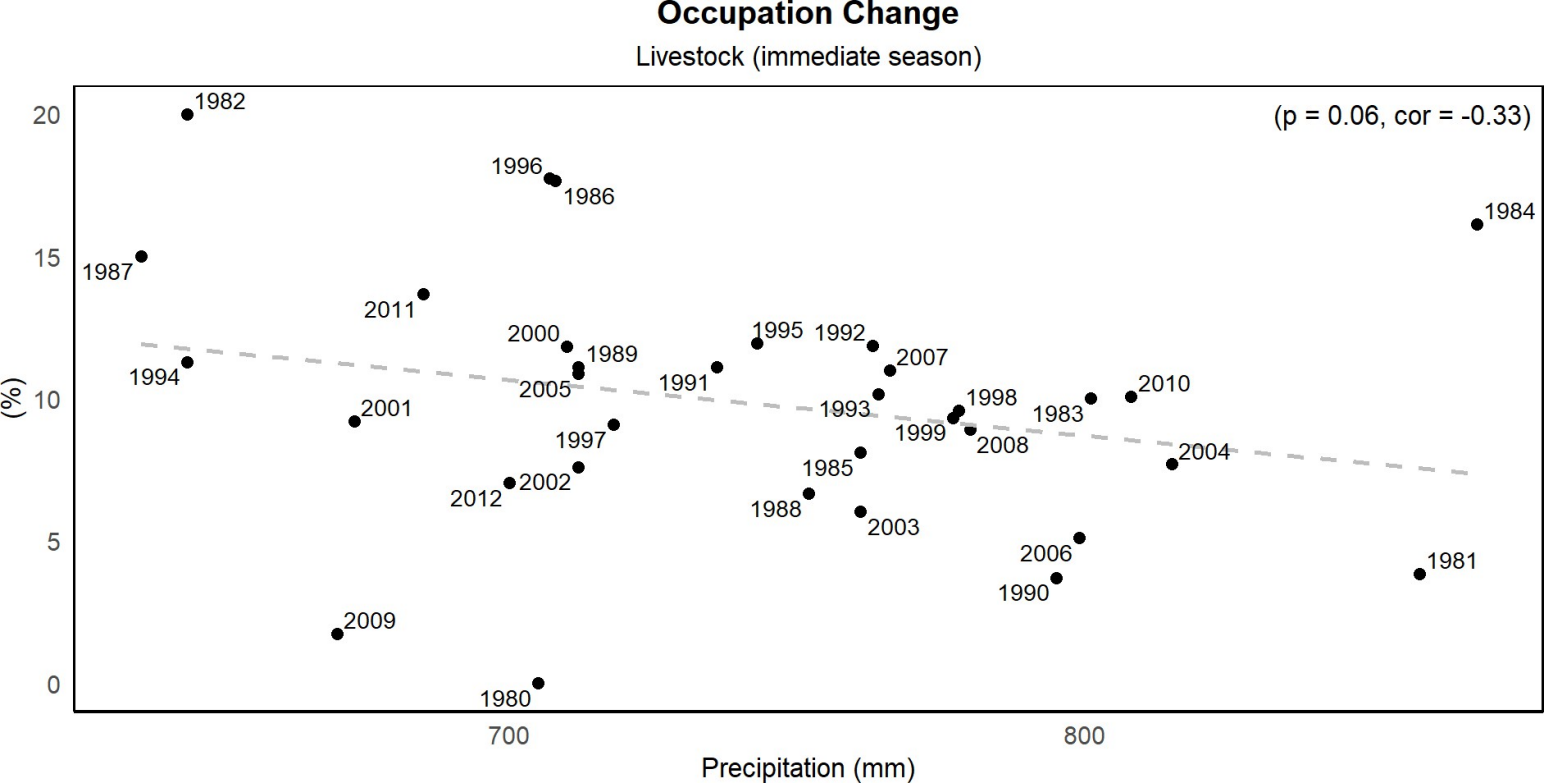
Livestock occupation immediate season. Correlation between mean annual precipitation and livestock raising occupation. The figure illustrates the immediate impact of drought on livestock herds, the lower the rainfall, the higher the number of workers who quit the livestock activity in any particular year. The Pearson correlation coefficient and significance test are presented in the top left part of the figure. (Source of data: calculations by the authors using EDER 2017 [50]).

We found a strong impact of precipitation variability on agrarian occupation, particularly for those working in rainfed agriculture and livestock raising. For the first case, we found that precipitation strongly influences the decision to remain in the activity in the subsequent year (Fig 5). In other words, the lower the rainfall, the higher the number of workers who quit the rainfed agriculture activity in the next year and vice versa. The highest rates of abandonment were observed in 1988 and 1983, with 12% and 9% of workers leaving the activity, respectively. These years followed the two driest on record. A similar pattern is observed for the livestock sector (Fig 6), but with impacts in the same year. We found a linear and negative relationship between occupation abandonment and precipitation variability (p = 0.06, r = -0.33). Thus, the lower the rainfall, the higher the number of workers who quit the livestock activity in any particular year. Like the previous case, the largest values (15 and 20%) correspond to the driest years on record, 1982 and 1987. Interestingly, we found that 31% of individuals who abandoned agricultural occupations, whether in rainfed agriculture or cattle ranching, became unemployed.

Additionally, a positive relationship of precipitation with planted cropland area was observed (p<0.05, cor = 0.19). This indicates that lower rainfall is associated with a smaller total planted area, which in turn affects income (p<0.05, cor = 0.18). On average, from 1980 to 2017, income decreased by 230,951 million pesos during dry years (i.e. those with precipitation 1sd below the average), while the total agricultural area decreased by 350,717 hectares, equivalent to approximately 3% of the total area.

#### 3.1.3 Long-term seasonal trends

**Fig 7:**
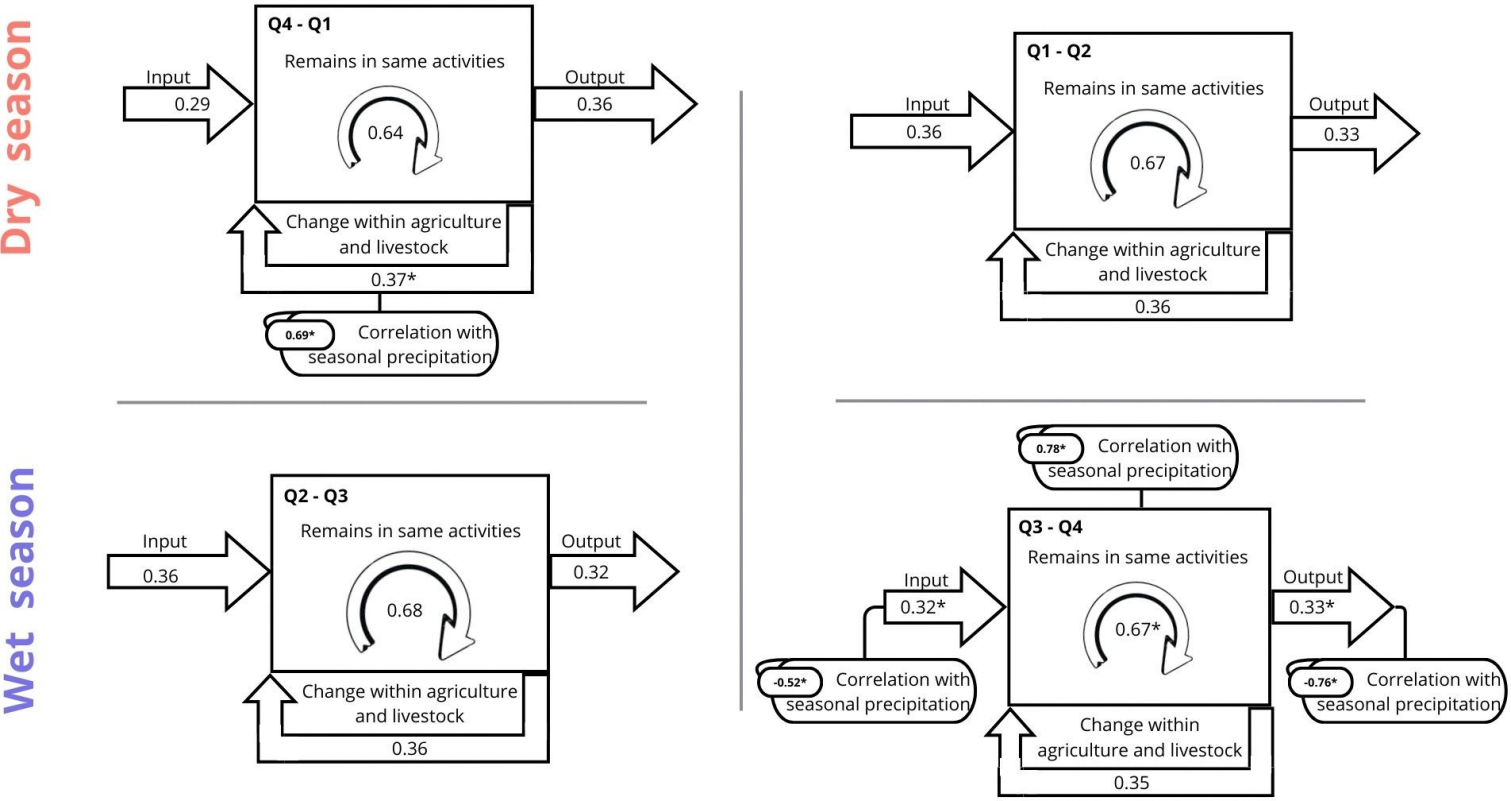
Correlation between seasonal rainfall and occupational change by quarter. The dynamics of farmers and livestock farmers exhibit variations in response to climatic variability throughout the year. Essentially, occupational patterns may shift according to the seasonality of rainfall. During the transition from the wet season to the dry season (a), respondents tend to change their occupation within the agricultural sector. Meanwhile, throughout the dry season and the commencement of the rainy season (b) most individuals tend to persist in the same activities. During the peak of the wet season (c), a significant proportion of workers return to their primary activities, maintaining their occupations. However, with increasing uncertainty in rainfall, particularly when there is a decrease, the probability of changing occupations rises (a). In summary, if rainfall increases (d), it is possible that agricultural inflows and outflows will increase, but the possibility of remaining in the same occupation will also increase. (Source of data: calculations by the authors using ENOE 2005–2017 [56]).

We study long-term changes in agrarian occupation in Mexico from 2005 to 2017 by dividing the time series into four-month periods (quarters) (Fig 7). In this way, each quarterly transition coincides with the beginning or end of the two main rainy seasons in the country. The transitions from Q4 to Q1 (fourth quarter to first quarter) not only reflects the shift from one year to the next but also marks the transition from the wet to the dry season (Fig 7A). During this transition, there is a decrease in people who start working in the agriculture (inputs) and an increase in the people who leave this sector (outputs). Nevertheless, most people employed in agriculture remained in the same activity. Interestingly, we found a positive correlation (r = 0.69, p<0.05) between rainfall and changes in activities within the agricultural sector (e.g. people shifting from rainfed agriculture to livestock raising). In other words, with an increase in rainfall the possibilities of changing economic activity in agriculture increased; for example, transitioning from cultivating crops to raising cattle, for a larger profit.

The transitions from Q1 to Q2 (first quarter to second quarter) (Fig 7B) marks the full extent of the dry season and the onset of the rainy season. Here inputs to the agrarian sector increase while outputs decrease, marked by the need for more people employed in agriculture as rainfall starts (e.g. to plow the fields). Interestingly, during this transition, precipitation variability seems to have little impact on demographic flows and occupation decisions; thus, the seasonal flux of people entering the activity seems to be unrelated to the amount of precipitation.

The transition between Q2 and Q3 (from the second quarter to the third quarter) (Fig 7C) represents the shift from the dry season to the beginning of the wet season of the year. The graph shows that during this transition, inputs to the agricultural sector are maintained while outputs decrease significantly. In other words, and considering the previous quarter, a large proportion of workers remain in their primary activities. Similar to the last transition, it is interesting to see that during this quarter, precipitation seems to have little impact on the fluxes, with no difference across years.

The last transition from Q3 to Q4 (from the third quarter to the fourth quarter) (Fig 7D) marks the end of the rainy season and is the period with the highest uncertainty in the rainfall (i.e. precipitation standard deviation is59 mm in the third quarter vs. 19 mm in the others). During this transition, inputs and outputs are mostly equivalent and highly dependent on rainfall, as well as the people remaining in the agrarian activities. For example, we found a positive correlation between rainfall and people staying in the same activity (r = 0.78, p<0.05): the more it rains, the higher the chances of remaining in the same activity. Likewise, we also found a relationship of precipitation with the inputs (r = -0.52, p = 0.05) and outputs (r = -0.76, p<0.05). Thus, precipitation amount and rainy season duration, determines how many people remain working in the agricultural sector, which may explain why during dry years there is a net loss of people occupied in agriculture.

## 4. Discussion

### 4.1 Long term agrarian occupation variability

Human societies are complex systems characterized by multiple patterns, structures, and properties that determine their demographic processes [63]. Therefore, deterministic narratives that suggest climate inevitably causes migration, violence, or ill health should be avoided [64]. Thus, climate should be conceived as one of several factors influencing decision-making in the face of potential challenges facing humans [65], and not as a delimiting factor. In that sense, the control of climate over agriculture is evident, but how this influences people’s decisions it’s mediated by a plethora of other factors. In the particular case of our study, climate seems to influence agrarian occupation, particularly during extreme dry years.

In the long term we found a steady decline of people engaged in agriculture in Mexico from 1980 to 2017, consistent across all datasets. This was also reported by Hernández Pérez [66], who estimated a loss of more than one million rural jobs (1,178,067) between 1995 and 2020. Similarly, Weisbrot et al. [67] reported a decrease of 1.9 million jobs, but the loss was in family labor employed in the family farm sector. Cypher and Crossa [68] argue that this phenomenon is an indication of the country’s agrarian policies, since Mexican manufacturing employment increased, but never counteracted the vertiginous reduction of labor and disarticulation of the peasant economy. Weisbrot et al. [67] further explain that this phenomenon develops because family farms in Mexico cannot compete with subsidized U.S. production. So, workers are displaced to other occupations, such as industry or seasonal jobs, including growing vegetables or fruits for export. Therefore, it is undeniable that there is research focused on explaining the decrease in Mexican agrarian occupation as a socioeconomic and political consequence. However, not much is known about the influence of climate, specifically the high variability in precipitation, on the decision making of farmers and livestock farmers.

We found that the average age at which individuals abandon agrarian occupations has consistently increased over time, indicating a gradual exit from agricultural work. Initially, younger people tend to leave first, followed by older individuals as we get closer to the present day. A study by the Food and Agriculture Organization of the United Nations (FAO) and the Secretariat of Agriculture and Rural Development (SADER) [69] revealed that by 2014, nearly 60% of the agricultural workforce in the country was over 50 years old. This is significant because older farmers may leave due to health issues or the presence of a successor, whereas younger farmers often exit for professional, lifestyle, and financial reasons [70]. Therefore, it would be incorrect to conclude that the abandonment of agrarian occupations is primarily driven by demographic age factors; instead, this aspect is secondary to other individual and household characteristics [71].

In addition, we identified the climate influence on the agrarian occupation in Mexico on two-time scales: first, across years, and secondly, throughout the year. These results align with Zhao et al. [72], who demonstrated that there are inter-annual variations in agricultural market yields as a consequence of the incidence of climate. However, this phenomenon is further magnified by endogenous market fluctuations generated by producers’ imperfect expectations about weather conditions.

#### 4.1.1 Interannual variability

In terms of interannual variability, our results suggest that rainfall influences the percentage of people engaged in rainfed agriculture and livestock in every year and the subsequent year. Similar relationships have been observed by other researchers. For example, Macfarlan et al. [73] found that livestock farmers in Baja California Sur adjust herd composition and usage during drought years, becoming more risk averse. The authors suggest that future expectations are important in the preference structure. Thus, individuals who expected to lose a substantial fraction of their herds during a drought were more likely to up-regulate herd maintenance at the expense of sales, and vice versa for those who expected smaller losses. Similarly, Freudenreich and Musshoff [74] observed that, in Chiapas, farmers engaged in maize cultivation may develop increased risk aversion if they have suffered severe and recurrent crop failures. This means that, after experiencing shocks such as droughts, they may overestimate themselves as they make agricultural decisions; for example, avoiding engaging in risky practices that could improve productivity or implementing adaptive behaviors that reduce risk. There is the possibility that one of these decisions is to migrate, since the reduction in the productivity of milpas or livestock reduces the income of rural farmers [24, 26, 75].

Furthermore we found that 31% of the Mexican population who leaves their agricultural occupations is unemployed in the upcoming year. Recent evidence from the U.S. suggests that this unemployment is due to limited strategies for adapting to and mitigating the impacts of climate change on agricultural yields [76]. In Mexico, such policies propose changes to ensure higher capital productivity; however, they generally do not favor labor substitution [77]. This implies that Mexican farmers do not have access to the same portfolio of adaptation strategies as U.S. farmers, likely placing them in a less favorable position to adapt to climate change [33]. Consequently, their livelihoods are not guaranteed. In contrast, countries like Algeria, Germany, and Romania have sought to ensure that workers affected by environmental risks are entitled to benefits for occupational accidents [78].

In addition, a correlation between rainfall and planted was observed. This aligns with the findings of Iizumi and Navin [79], who stated that both climate and weather conditions influence the area, intensity, and yield of crops. Similarly, studies such as those by Koide et al. [80] and Naylor et al. [81] demonstrate a strong dependence of rice-planted areas on accumulated rainfall in the Philippines and Indonesia. Thus, during dry years smaller agricultural area is available for crops, needing less workers, reducing employment.

Additionally, our results also show that low precipitation leads to a decrease in agricultural income during dry years. In this regard, Bobojonov and Aw-Hassan’s [82] research suggests that this is due to the risks associated with extreme weather events, as they found a negative impact on farmers’ income in small-scale farms in arid regions of Tajikistan when the availability of irrigation water decreased. As for research in the Mexican context, there remains a gap in data exploring the correlation between climate variables, sown area, and economic income. However, Boyd and Ibarrarán [83] note that the effects of droughts or hurricanes on the country’s economy vary substantially by sector, though the overall impacts tend to be moderate to severe.

#### 4.1.2 Long-term seasonal trends

In terms of seasonal variability, we found that precipitation drives seasonal changes in employment, with a higher employment chance during the rainy season and vice versa. If rainfall is greater in duration and quantity, more people will enter the agricultural sector and fewer will leave. Additionally, they are likely to maintain their original occupation due to the potential benefits of the activity, whereas the opposite occurs during dry years. These finding are consistent with research such as Estrada et al. [20], which argues that agriculture, especially rainfed agriculture, is highly dependent on predictable precipitation. Thus, if rainfall becomes less predictable in timing and quantity, crop needs may not be met, leading to lower yields and higher food prices [84]. Therefore, knowledge of precipitation trends is of utmost importance for sowing and harvesting periods [85]. For example, excessive sunshine can accelerate fruit ripening, making the yield inversely proportional to the harvesting time and resulting in an excess labor force, which facilitates piecework payment [86]. Similarly, in livestock management, farmers make decisions considering that annual variability in precipitation is responsible for the large variation in forage available on pasture [22, 87]. In this context, adaptive strategies throughout human evolutionary history have been related to seasonality [88].

Finally, our results also imply that climate change, particularly increasing drying conditions, may lead to higher rates of agricultural abandonment in Mexico. According to the Intergovernmental Panel on Climate Change (IPCC) [89], Mexico could experience statistically significant reductions in gross domestic product growth (GDP), especially in agricultural activities, as a result of current temperatures being above the threshold considered optimal for economic production. Estrada et al. [20] clarify that the impacts of climate change on Mexican agriculture develop directly in local economies, but with impacts across the whole country in the most extreme scenarios. Thus, the link between climate change and agricultural abandonment will depend on the alteration of precipitation patterns and the fact that high-exposure working conditions for farmers and livestock farmers imply greater heat stress [90]. Additionally, Dasgupta et al. [91] mention that current climatic conditions already negatively affect labor effectiveness; however, climate change may further exacerbate the decline in productivity and labor supply. This is due to reduced individual income and the need to adapt working hours to avoid exposure to harsh climate conditions.

#### 4.1.3 Policy implications

Climate variability is widely recognized for its significant impact on crop yields, directly influencing food production [20]. Numerous studies have also highlighted the effects of climate fluctuations on populations [92]. This article demonstrates that in Mexico, rainfall variability plays a key role in shaping decision-making and the mobility of individuals engaged in agrarian occupations.

This information is vital for developing national agricultural management strategies that address the challenges posed by climate variability. One of the greatest challenges is the uncertainty surrounding future climate conditions and their impact on crop yields, which could exceed the adaptive capacity of workers, thereby threatening their livelihoods [92]. Therefore, it is crucial to begin mitigating potential damage to the food value chain [93] and to increase investment in agricultural workers. This is especially important given that these workers often rely on additional labor rather than capital, as farmers are frequently constrained by limited cash and credit for growing and harvesting [94].

Policymakers should also focus on reducing social vulnerability to minimize the risks posed by extreme weather events, such as drought. Regarding agricultural impacts, the primary policy focus should be on water management [95]. For instance, one strategy could be to reduce inequality in access to public services, which facilitates workers’ adaptation [96]. However, to cope with an increasingly unpredictable climate, there is a need for more efficient and integrated water management systems at the local level, including the development of technologies for water storage [95]. In this context, it is recommended that decision-makers pay close attention to individuals in agrarian occupations, as their knowledge and experience complement scientific information in the formulation of policies and programs. These programs should aim to improve and introduce technologies appropriate to the region’s biophysical context and align with the interests of the population [94]. Such an approach could lead to the design of initiatives that inform and support planting decisions while providing financial assistance for mechanization and the construction of adequate irrigation systems [94].

## 5. Conclusion

Our analysis supports a link between rainfall and agrarian occupation in Mexico from 1980 and 2017. We find that reduced rainfall correlates with a greater increase in changes in agrarian occupations; however, the impact varies among different agricultural workers. Cattle livestock farmers often change occupations during drought years, whereas, in agriculture, changes in occupation are observed predominantly among those relying on rainfed methods. Our research further demonstrates that precipitation levels have significantly influenced the decision-making and migratory patterns of both crop and livestock farmers. However, this phenomenon is not solely an interannual occurrence; it also has seasonal and quarterly repercussions.

The study of the link between precipitation and agrarian occupations highlights the broader effects of climate change on agricultural practices, labor markets, and food security in regions heavily dependent on rainfed agriculture. While this analysis provides valuable insights into how climate variability affects agricultural occupations, it has limitations. Specifically, it operates on a national scale, which may obscure localized effects and fails to fully account for regional knowledge, diverse practices, and the varying climatic and socioeconomic conditions across different areas. Additionally, data prior to 1990 are limited, so the findings should be interpreted with caution.

To avoid overgeneralizing these results, future research should focus on exploring the socioeconomic backgrounds of the agrarian labor force at the local level, as well as the climate conditions specific to each region. This approach would help capture the negative effects of climate variability in different contexts. Furthermore, more detailed research on the economic activities in which individuals engage is needed to analyze transitions between agrarian work and other sectors. Studies are also required to examine the interactions between climatic variables, such as temperature and precipitation, and their impacts on agricultural productivity, particularly across different farming systems. While the current analysis could benefit from a closer look at specific farming systems and the varying magnitude of climate variability’s effects, the available data consist of national aggregates that lack detailed regional development. Addressing these gaps in future research would offer a more comprehensive understanding of how climate influences agricultural livelihoods and could inform policy aimed at improving resilience to changing climate conditions.

## Supporting information

S1 Fig Average age of occupation abandonment. We also observed that the average age of departure from agrarian occupations shows a consistent increment. For example, between 1980 and 1990, it ranged between 15 and 19 years, whereas from 2000 to 2017, it fluctuated between 23 and 35 years. This trend suggests a gradual abandonment of agrarian occupations, where younger individuals left first and are recently followed by older individuals (S1 Fig).
(TIF)

## References

[1] Iizumi T, Ramankutty N. Changes in yield variability of major crops for 1981–2010 explained by climate change. Environ Res Lett [Internet]. 2016;11(3):034003. Available from: 10.1088/1748-9326/11/3/034003

[2] Sultan B, Defrance D, Iizumi T. Evidence of crop production losses in West Africa due to historical global warming in two crop models. Sci Rep [Internet]. 2019;9(1). Available from: doi: 10.1038/s41598-019-49167-0 31492929 PMC6731230

[3] Moura da Silva EH, Silva Antolin LA, Zanon AJ, Soares Andrade A Junior, Antunes de Souza H, dos Santos Carvalho K, et al. Impact assessment of soybean yield and water productivity in Brazil due to climate change. Eur J Agron [Internet]. 2021;129(126329):126329. Available from: 10.1016/j.eja.2021.126329

[4] Ciais P, Reichstein M, Viovy N, Granier A, Ogée J, Allard V, et al. Europe-wide reduction in primary productivity caused by the heat and drought in 2003. Nature [Internet]. 2005;437(7058):529–33. Available from: https://www.nature.com/articles/nature03972 doi: 10.1038/nature03972 16177786

[5] Beillouin D, Schauberger B, Bastos A, Ciais P, Makowski D. Impact of extreme weather conditions on European crop production in 2018. Philos Trans R Soc Lond B Biol Sci [Internet]. 2020;375(1810):20190510. Available from: doi: 10.1098/rstb.2019.0510 32892735 PMC7485097

[6] Olmstead AL, Rhode PW. Adapting North American wheat production to climatic challenges, 1839–2009. Proc Natl Acad Sci U S A [Internet]. 2011;108(2):480–5. Available from: doi: 10.1073/pnas.1008279108 21187376 PMC3021086

[7] Zipper SC, Qiu J, Kucharik CJ. Drought effects on US maize and soybean production: spatiotemporal patterns and historical changes. Environ Res Lett [Internet]. 2016;11(9):094021. Available from: 10.1088/1748-9326/11/9/094021

[8] Pearson LJ, Nelson R, Crimp S, Langridge J. Interpretive review of conceptual frameworks and research models that inform Australia’s agricultural vulnerability to climate change. Environ Model Softw [Internet]. 2011;26(2):113–23. Available from: 10.1016/j.envsoft.2010.07.001

[9] Dun O, Klocker N, Farbotko C, McMichael C. Climate change adaptation in agriculture: Learning from an international labour mobility programme in Australia and the Pacific Islands region. Environ Sci Policy [Internet]. 2023;139:250–73. Available from: 10.1016/j.envsci.2022.10.017

[10] Liu Q, Shen B, Wen X. Role of climate-smart agriculture in fighting against climate change in competitive supply chains. Int J Prod Econ [Internet]. 2023;264(108978):108978. Available from: 10.1016/j.ijpe.2023.108978

[11] Colin KP, Mohtadi S, Cane MA, Seager R, Kushnir Y. Climate change in the Fertile Crescent and implications of the recent Syrian drought. Proc Natl Acad Sci U S A [Internet]. 2015;112(11):3241–6. Available from: doi: 10.1073/pnas.1421533112 25733898 PMC4371967

[12] O’Loughlin J, Linke AM, Witmer FDW. Effects of temperature and precipitation variability on the risk of violence in sub-Saharan Africa, 1980–2012. Proc Natl Acad Sci U S A [Internet]. 2014;111(47):16712–7. Available from: doi: 10.1073/pnas.1411899111 25385621 PMC4250158

[13] Randell H, Gray C. Climate change and educational attainment in the global tropics. Proc Natl Acad Sci U S A [Internet]. 2019;116(18):8840–5. Available from: doi: 10.1073/pnas.1817480116 30988183 PMC6500158

[14] Kono DY. Compensating for the climate: Unemployment insurance and climate change votes. Polit Stud [Internet]. 2020;68(1):167–86. Available from: 10.1177/0032321719836066

[15] Montt G, Fraga F. The future of work in a changing natural environment: Climate change, degradation and sustainability [Internet]. Ilo.org. 2018. Available from: https://www.ilo.org/sites/default/files/wcmsp5/groups/public/@dgreports/@cabinet/documents/publication/wcms_644145.pdf

[16] Liu T-Y, Lin Y. Does global warming affect unemployment? International evidence. Econ Anal Policy [Internet]. 2023;80:991–1005. Available from: 10.1016/j.eap.2023.09.028

[17] Alehile KS. Climate change effects on employment in the Nigeria’s agricultural sector. Chin J Urban Environ Stud. 2023;11(03). Available from: 10.1142/s2345748123500185

[18] Pontuti TA. Drought and Agricultural Employment in the United States [Internet]. Vol. 30570807. 2023. Available from: https://www.proquest.com/docview/2877309758?pq-origsite=gscholar&fromopenview=true&sourcetype=Dissertations%20&%20Theses

[19] Acevedo I, Castellani F, Lopez de la Cerda C, Lotti G, Székely M. Natural disasters and labor market outcomes in Mexico. Inter-American Development Bank; 2023.

[20] Estrada F, Mendoza-Ponce A, Calderón-Bustamante O, Botzen W. Impacts and economic costs of climate change on Mexican agriculture. Reg Environ Change [Internet]. 2022;22(4). Available from: 10.1007/s10113-022-01986-0

[21] Challenger A. Utilización y conservación de los ecosistemas terrestres de México: pasado presente y futuro [Internet]. Gob.mx. 1998. Available from: http://www.conabio.gob.mx/institucion/proyectos/resultados/FichapubE019.pdf

[22] Murray-Tortarolo GN, Jaramillo VJ. Precipitation extremes in recent decades impact cattle populations at the global and national scales. Sci Total Environ [Internet]. 2020;736(139557):139557. Available from: doi: 10.1016/j.scitotenv.2020.139557 32473457

[23] Murray-Tortarolo GN, Jaramillo VJ. The impact of extreme weather events on livestock populations: the case of the 2011 drought in Mexico. Clim Change [Internet]. 2019;153(1–2):79–89. Available from: 10.1007/s10584-019-02373-1

[24] Feng S, Krueger AB, Oppenheimer M. Linkages among climate change, crop yields and Mexico–US cross-border migration. Proc Natl Acad Sci U S A [Internet]. 2010;107(32):14257–62. Available from: doi: 10.1073/pnas.1002632107 20660749 PMC2922556

[25] Hunter LM, Murray S, Riosmena F. Rainfall patterns and U.s. migration from rural Mexico. Int Migr Rev [Internet]. 2013;47(4):874–909. Available from: doi: 10.1111/imre.12051 25473143 PMC4243932

[26] Murray-Tortarolo GN, Salgado MM. Drought as a driver of Mexico-US migration. Clim Change [Internet]. 2021;164(3–4). Available from: 10.1007/s10584-021-03030-2

[27] United Nations Conference on Trade and Development (UNCTAD). Mexico’s agriculture development: Perspectives and outlook [Internet]. Unctad.org. 2013. Available from: https://unctad.org/system/files/official-document/ditctncd2012d2_en.pdf

[28] Sánchez MV, Cicowiez M, Ortega A. Prioritizing public investment in agriculture for post-COVID-19 recovery: A sectoral ranking for Mexico. Food Policy [Internet]. 2022;109(102251):102251. Available from: 10.1016/j.foodpol.2022.102251

[29] Negrete JC, Kriuskova ER, De Jesus Lopez Canteñs G, Avila CIZ, Hernandez GL. Arduino Board in the Automation of Agriculture in Mexico, A review. Int J Hortic [Internet]. 2018;8(0). Available from: https://hortherbpublisher.com/index.php/ijh/article/view/3452

[30] Villanueva L, Jiang X. Export collapse and employment effects during the Covid-19 crisis in Mexico. Probl Desarro [Internet]. 2022;53(210). Available from: 10.22201/iiec.20078951e.2022.210.69842

[31] Morton JF. The impact of climate change on smallholder and subsistence agriculture. Proc Natl Acad Sci U S A [Internet]. 2007;104(50):19680–5. Available from: doi: 10.1073/pnas.0701855104 18077400 PMC2148357

[32] Rathi A. Is Agrarian Resilience limited to Agriculture? Investigating the “farm” and “non-farm” processes of Agriculture Resilience in the rural. J Rural Stud [Internet]. 2022;93:155–64. Available from: 10.1016/j.jrurstud.2019.12.015

[33] Jessoe K, Manning DT, Taylor JE. Climate change and labour allocation in rural Mexico: Evidence from annual fluctuations in weather. Econ J (London) [Internet]. 2018;128(608):230–61. Available from: 10.1111/ecoj.12448

[34] Blaise J, Fairclough M. Mexico Geography Geology [Internet]. Iaea.org. 2020. Available from: https://infcis.iaea.org/udepo/Resources/Countries/Mexico.pdf

[35] Delgado GC, Gay García C, Ímaz Gispert MA, Martínez MA, Ímaz Gispert MA, Blazquez Graf N, et al. México frente al cambio climático: retos y oportunidades [Internet]. Edu.ar. 2013. Available from: https://biblioteca.clacso.edu.ar/Mexico/ceiich-unam/20170502052756/pdf_1468.pdf

[36] Rodríguez-Moreno VM, Medina-García G, Padilla GD, Corral JA-R, Estrada-Avalos J, Ruvalcaba JEM. ¿Por qué México es un país altamente vulnerable al cambio climático? [Internet]. Gob.mx. 2021. Available from: https://cienciasagricolas.inifap.gob.mx/index.php/agricolas/article/download/2819/4550/23113

[37] PNUD-INECC. Impactos de la elevación del nivel del mar en ecosistemas y especies de 35 islas pobladas y prioritarias de México. 2017; 29. Available from: https://datos.abiertos.inecc.gob.mx/Datos_abiertos_INECC/CGACC/DocumentosRIslasMarias/Eje3_ImpactosDelCambioClimaticoEnTerritorioInsularMexicano/EstudiosAguirreEtAl/IslasMarEcosistemas.pdf

[38] Murray-Tortarolo GN. Seven decades of climate change across Mexico. Atmósfera [Internet]. 2021; Available from: https://www.redalyc.org/articulo.oa?id=56572300007

[39] Priego Santander AG, Esteve Selma MA. Análisis de la complejidad y heterogeneidad de los paisajes de México [Internet]. Redalyc.org. 2017. Available from: https://www.redalyc.org/pdf/407/40751261001.pdf

[40] Cervantes Martínez A. Presentación. Teoría y Praxis. Teoría y Praxis [Internet]. 2016;(19):9. Available from: https://www.redalyc.org/articulo.oa?id=456146535001

[41] Morrone JJ. Regionalización biogeográfica y evolución biótica de México: encrucijada de la biodiversidad del Nuevo Mundo. Revista Mexicana de Biodiversidad [Internet]. Unam.mx. 2019. Available from: http://rev.mex.biodivers.unam.mx/index.php/es/encrucijada-de-la-biodiversidad/

[42] Sen AK. Desarrollo y Libertad. Planeta; 2000.

[43] Consejo Nacional de Evaluación de la Política de Desarrollo Social (CONEVAL). La pobreza por ingresos en México [Internet]. Org.mx. 2010. Available from: https://www.coneval.org.mx/rw/resource/coneval/info_public/pdf_publicaciones/pobreza_ingresos_mexico_web.pdf

[44] Negrete M. La distribución del ingreso y la riqueza: nuevas aproximaciones conceptuales y metodológicas. Síntesis [Internet]. 2023. Available from: https://www.cepal.org/es/publicaciones/48636-la-distribucion-ingreso-la-riqueza-nuevas-aproximaciones-conceptuales

[45] Consejo Nacional de Evaluación de la Política de Desarrollo Social (CONEVAL). 10 Años de medición de pobreza en México, Avances y retos en política social [Internet]. Org.mx. 2019. Available from: https://www.coneval.org.mx/SalaPrensa/Comunicadosprensa/Documents/2019/COMUNICADO_10_MEDICION_POBREZA_2008_2018.pdf

[46] Consejo Nacional de Evaluación de la Política de Desarrollo Social (CONEVAL). Documento de Análisis sobre la Medición Multidimensional de la Pobreza, 2022 [Internet]. Org.mx. 2022. Available from: https://www.coneval.org.mx/Medicion/MP/Documents/MMP_2022/Documento_de_analisis_sobre_la_medicion_multidimensional_de_la_pobreza_2022.pdf

[47] Servicio de Información Agroalimentaria y Pesquera (SIAP). Panorama agroalimentario 2019. Servicio de Información Agroalimentaria y Pesquera, Secretaría de Agricultura y Desarrollo Rural, México. 2019. Available from: https://www.gob.mx/siap/es/articulos/panorama-agroalimentario-2019-nos-muestra-la-realidad-del-sector-y-tambien-nos-deja-ver-su-enorme-potencial?idiom=es

[48] Instituto Nacional de Estadística y Geografía (INEGI). Cómo se hace la ENOE. Métodos y procedimientos [Internet]. Org.mx. 2020. Available from: https://www.inegi.org.mx/app/biblioteca/ficha.html?upc=702825190613

[49] Harris I, Osborn TJ, Jones P, Lister D. Version 4 of the CRU TS monthly high-resolution gridded multivariate climate dataset. Scientific data, 7(1), 1–18. Available from: https://crudata.uea.ac.uk/cru/data/hrg/cru_ts_4.06/crucy.2205251923.v4.06/countries/pre/10.1038/s41597-020-0453-3 PMC7125108 32246091

[50] Instituto Nacional de Estadística y Geografía (INEGI). Encuesta Demográfica Retrospectiva (EDER) 2017 [Data file]. Org.mx. 2017. Available from: https://www.inegi.org.mx/programas/eder/2017/

[51] Instituto Nacional de Estadística y Geografía (INEGI). Encuesta Demográfica Retrospectiva 2017 EDER: Marco conceptual [Internet]. Org.mx. 2018. Available from: https://www.inegi.org.mx/contenidos/productos/prod_serv/contenidos/espanol/bvinegi/productos/nueva_estruc/702825103590.pdf

[52] Instituto Nacional de Estadística y Geografía (INEGI) (2014). La informalidad laboral. Marco conceptual y metodológico. Org.mx. 2014. Available from: https://www.snieg.mx/DocAcervoINN/documentacion/inf_nvo_acervo/SNIDS/ENOE/702825060459.pdf

[53] Escoto AR. ¿Cómo empezar a estudiar el mercado de trabajo en México? [Internet]. Comecso.com. 2022. Available from: https://www.comecso.com/observatorio/estudiar-mercado-de-trabajo-mexico

[54] Instituto Nacional de Estadística y Geografía (INEGI). Encuesta Nacional de Empleo (ENE) [Data file]. Available from: Org.mx. https://www.inegi.org.mx/programas/ene/2004/

[55] Instituto Nacional de Estadística y Geografía (INEGI). Estadísticas sobre la dinámica laboral en México 2000–2004 [Internet]. Org.mx. 2006. Available from: https://www.inegi.org.mx/app/biblioteca/ficha.html?upc=702825444174

[56] Instituto Nacional de Estadística y Geografía (INEGI). Encuesta Nacional de Ocupación y Empleo (ENOE) población de 15 años y más de edad [Data file]. Org.mx. 2005–2017. Available from: https://www.inegi.org.mx/programas/enoe/15ymas/.

[57] Cámara de Diputados del H Congreso de la Unión. Political Constitution of the United Mexican States [Internet]. Gob.mx. [cited 2024 Nov 13]. Available from: https://www.oas.org/ext/Portals/33/Files/Member-States/Mex_intro_txtfun_eng.pdf

[58] Cámara de Diputados del H Congreso de la Unión. Ley Federal de Protección de Datos Personales en Posesión de los Particulares [Internet]. Gob.mx. [cited 2024 Nov 13]. Available from: https://www.diputados.gob.mx/LeyesBiblio/pdf/LFPDPPP.pdf

[59] Instituto Nacional de Estadística y Geografía (INEGI). Sistema nacional de clasificación de ocupaciones 2011 [Internet]. Snieg.mx 2011. Available from: https://www.snieg.mx/DocumentacionPortal/Normatividad/historica/sinco-2012.pdf

[60] Instituto Nacional de Estadística y Geografía (INEGI). ENOE. Clasificación Mexicana de Ocupaciones (CMO) [Internet]. Org.mx. 2009. Volumen I. Org.mx. Available from: https://www.inegi.org.mx/contenidos/productos/prod_serv/contenidos/espanol/bvinegi/productos/metodologias/est/cmo_vol1.pdf

[61] Servicio de Información Agroalimentaria y Pesquera (SIAP). Statistical Yearbook of Agricultural Production [Data file]. Org.mx. Available from: https://nube.siap.gob.mx/cierreagricola/

[62] Instituto Nacional de Estadística y Geografía (INEGI). National Consumer Price Index [Data file]. Org.mx. Available from: https://www.inegi.org.mx/app/indicesdeprecios/CalculadoraInflacion.aspx

[63] Sawyer KR. Social emergence: Societies as complex systems. Contemporary Sociology [Internet]. Academia.edu. 2005. Available from: https://www.academia.edu/4512016/social_emergence_societies_as_complex_systems

[64] Robbins Schug G, Buikstra JE, DeWitte SN, Baker BJ, Berger E, Buzon MR, et al. Climate change, human health, and resilience in the Holocene. Proc Natl Acad Sci U S A [Internet]. 2023;120(4). Available from: doi: 10.1073/pnas.2209472120 36649426 PMC9942907

[65] Sherbinin A. Climate Impacts as Drivers of Migration [Internet]. Migrationpolicy.org. Available from: https://www.migrationpolicy.org/article/climate-impacts-drivers-migration87.

[66] Hernández Pérez JL. La agricultura mexicana del TLCAN al TMEC: consideraciones teóricas, balance general y perspectivas de desarrollo. El Trimestre [Internet]. 2021;88(352):1121–52. Available from: 10.20430/ete.v88i352.1274

[67] Weisbrot M, Merling L, Mello V, Lefebvre S, Sammut J. An Update After 23 Years [Internet]. Cepr.net. 2017. Available from: https://cepr.net/images/stories/reports/nafta-mexico-update-2017-03.pdf?v=2

[68] Cypher JM, Crossa M. T-MEC en el espejo del TLCAN: Engañosas ilusiones, brutales realidades. Ola Financ [Internet]. 2019;12(34). Available from: 10.22201/fe.18701442e.2019.34.71957

[69] FAO SADER. Estudio sobre el envejecimiento de la población rural en México [Internet]. Gob.mx. 2014. Available from: https://www.agricultura.gob.mx/sites/default/files/sagarpa/document/2019/01/28/1608/01022019-2-estudio-sobre-el-envejecimiento-de-la-poblacion-rural-en-mexico.pdf

[70] Brown P, Daigneault A, Dawson J. Age, values, farming objectives, past management decisions, and future intentions in New Zealand agriculture. J Environ Manage [Internet]. 2019;231:110–20. Available from: doi: 10.1016/j.jenvman.2018.10.018 30340130

[71] Burton RJF. An alternative to farmer age as an indicator of life-cycle stage: The case for a farm family age index. J Rural Stud [Internet]. 2006;22(4):485–92. Available from: 10.1016/j.jrurstud.2006.02.005

[72] Zhao X, Calvin KV, Wise MA, Patel PL, Snyder AC, Waldhoff ST, et al. Global agricultural responses to interannual climate and biophysical variability [Internet]. Iop.org. 2021. Available from: https://iopscience.iop.org/article/10.1088/1748-9326/ac2965/meta

[73] Macfarlan SJ, Schacht R, McCool WC, Davis C, Yerman A, Landeros FJH, et al. Decision-making under climate shocks and economic insecurity: Ranching in rural Baja California Sur, Mexico. Evol Hum Behav [Internet]. 2023;44(5):515–23. Available from: 10.1016/j.evolhumbehav.2023.07.001

[74] Freudenreich H, Musshoff O. Experience of losses and aversion to uncertainty—experimental evidence from farmers in Mexico. Ecol Econ [Internet]. 2022;195(107379):107379. Available from:: 10.1016/j.ecolecon.2022.107379

[75] Moreno Ramos RN. Family labour organization for dairy farming in western Mexico. Between the search for productivity and wellbeing. J Rural Stud [Internet]. 2021;88:354–67. Available from: 10.1016/j.jrurstud.2021.08.005

[76] Burke M, Emerick K. Adaptation to climate change: Evidence from US agriculture. Am Econ J Econ Policy [Internet]. 2016;8(3):106–40. Available from: 10.1257/pol.20130025

[77] de la Fuente Meraz A, Villarroel S. Impactos Socioeconómicos del Cambio Climático en México [Internet]. Uam.mx. 2018. Available from: http://dccd.cua.uam.mx/libros/investigacion/cambio_climatico_impactos_socioeconomicos.pdf

[78] International Labour Office (ILO). Working on a warmer planet: The impact of heat stress on labour productivity and decent work [Internet]. Ilo.org. 2019. Available from: https://www.ilo.org/sites/default/files/wcmsp5/groups/public/@dgreports/@dcomm/@publ/documents/publication/wcms_711919.pdf

[79] Iizumi T, Ramankutty N. How do weather and climate influence cropping area and intensity? Glob Food Sec [Internet]. 2015;4:46–50. Available from: 10.1016/j.gfs.2014.11.003

[80] Koide N, Robertson AW, Ines AVM, Qian J-H, DeWitt DG, Lucero A. Prediction of rice production in the Philippines using seasonal climate forecasts. J Appl Meteorol Climatol [Internet]. 2013;52(3):552–69. Available from: 10.1175/jamc-d-11-0254.1

[81] Naylor RL, Falcon WP, Rochberg D, Wada N. Clim Change [Internet]. 2001;50(3):255–65. Available from: 10.1023/a:1010662115348

[82] Bobojonov I, Aw-Hassan A. Impacts of climate change on farm income security in Central Asia: An integrated modeling approach. Agric Ecosyst Environ [Internet]. 2014;188:245–55. Available from: 10.1016/j.agee.2014.02.033

[83] Boyd R, Ibarrarán ME. Extreme climate events and adaptation: an exploratory analysis of drought in Mexico. Environ Dev Econ [Internet]. 2009;14(3):371–95. Available from: 10.1017/s1355770x08004956

[84] Schmidhuber J, Tubiello FN. Global food security under climate change. Proc Natl Acad Sci U S A [Internet]. 2007;104(50):19703–8. Available from: doi: 10.1073/pnas.0701976104 18077404 PMC2148361

[85] Rojas M, Lambert F, Ramirez-Villegas J, Challinor AJ. Emergence of robust precipitation changes across crop production areas in the 21st century. Proc Natl Acad Sci U S A [Internet]. 2019;116(14):6673–8. Available from: doi: 10.1073/pnas.1811463116 30858318 PMC6452695

[86] Sánchez-Gómez MJ, Lara-Flores SM. Los programas de trabajadores agrícolas temporales [Internet]. Unam.mx. 2015. Available from: https://ru.iis.sociales.unam.mx/bitstream/IIS/5229/1/progr_trabaj_agricolas.pdf

[87] Hart RH, Norton BE. Grazing management and vegetation response. In Vegetation science applications for rangeland analysis and management. (1988); (pp. 493–525). Springer Netherlands.

[88] Lee-Thorp J, Sponheimer M. Contribution of stable light isotopes to Paleoenvironmental reconstruction [Internet]. Scopus.com. 2015. Available from: https://www.scopus.com/record/display.uri?eid=2-s2.0-84944625509&origin=inward&txGid=91f5a7f7927f388aeaeb7927d9bbccb5

[89] Intergovernmental Panel on Climate Change (IPCC).Climate Change and Land: an IPCC special report on climate change, desertification, land degradation, sustainable land management, food security, and greenhouse gas fluxes in terrestrial ecosystems [Shukla P.R, Skea, Calvo Buendia E, Masson-Delmotte V., Pörtner H.-O, Roberts D. C, Zhai P, Slade R, Connors S., van Diemen R, Ferrat M, Haughey E., Luz S, Neogi S, Pathak M, Petzold J, Portugal Pereira J, Vyas P, Huntley E, Kissick K, Belkacemi M, Malley J]. 2019. In press, p. 258.

[90] Day E, Fankhauser S, Kingsmill N, Costa H, Mavrogianni A. Upholding labour productivity under climate change: an assessment of adaptation options. Clim Policy [Internet]. 2019;19(3):367–85. Available from: 10.1080/14693062.2018.1517640

[91] Dasgupta S, van Maanen N, Gosling SN, Piontek F, Otto C, Schleussner C-F. Effects of climate change on combined labour productivity and supply: an empirical, multi-model study. Lancet Planet Health [Internet]. 2021;5(7):e455–65. Available from: doi: 10.1016/S2542-5196(21)00170-4 34245716

[92] Sosa Rodríguez FS, Constantino Toto RM. Sequía en México [Internet]. Uam.mx. 2023. Available from: https://redaguam.xoc.uam.mx/wp-content/uploads/2023/08/Red-AgUAM-Sequia.pdf

[93] Calatayud A, Díez CF, De Groot R. Gestión de riesgos en cadenas de valor: Guía para el diseño de programas [Internet]. Iadb.org. 2017. Available from: https://publications.iadb.org/publications/spanish/document/Gesti%C3%B3n-de-riesgos-en-cadenas-de-valor-Gu%C3%ADa-para-el-dise%C3%B1o-de-programas.pdf

[94] Mardero S, Schmook B, Radel C, Christman Z, Lawrence D, Millones M, et al. Smallholders’ adaptations to droughts and climatic variability in southeastern Mexico. Environ Hazards [Internet]. 2015;14(4):271–88. Available from: 10.1080/17477891.2015.1058741

[95] Bozzola M, Swanson T. Policy implications of climate variability on agriculture: Water management in the Po river basin, Italy. Environ Sci Policy [Internet]. 2014;43:26–38. Available from: 10.1016/j.envsci.2013.12.002

[96] Moreno MGC, Rueda EC. Situación de desigualdad en el acceso al agua y saneamiento de la región hidrosocial-política e intercultural de Las Margaritas y La Trinitaria, Chiapas, México [Internet]. Unam.mx. 2023. Available from: https://ru.iiec.unam.mx/6268/1/2.%20239-Corona-Cruz.pdf

