# Supplementary figures and images for: The impact of climate variability on agricultural employment in Mexico from 1980–2017
Source: PLoS One. 2025 Feb 10;20(2):e0313891. doi: 10.1371/journal.pone.0313891 (PMC11809791; doi:10.1371/journal.pone.0313891)

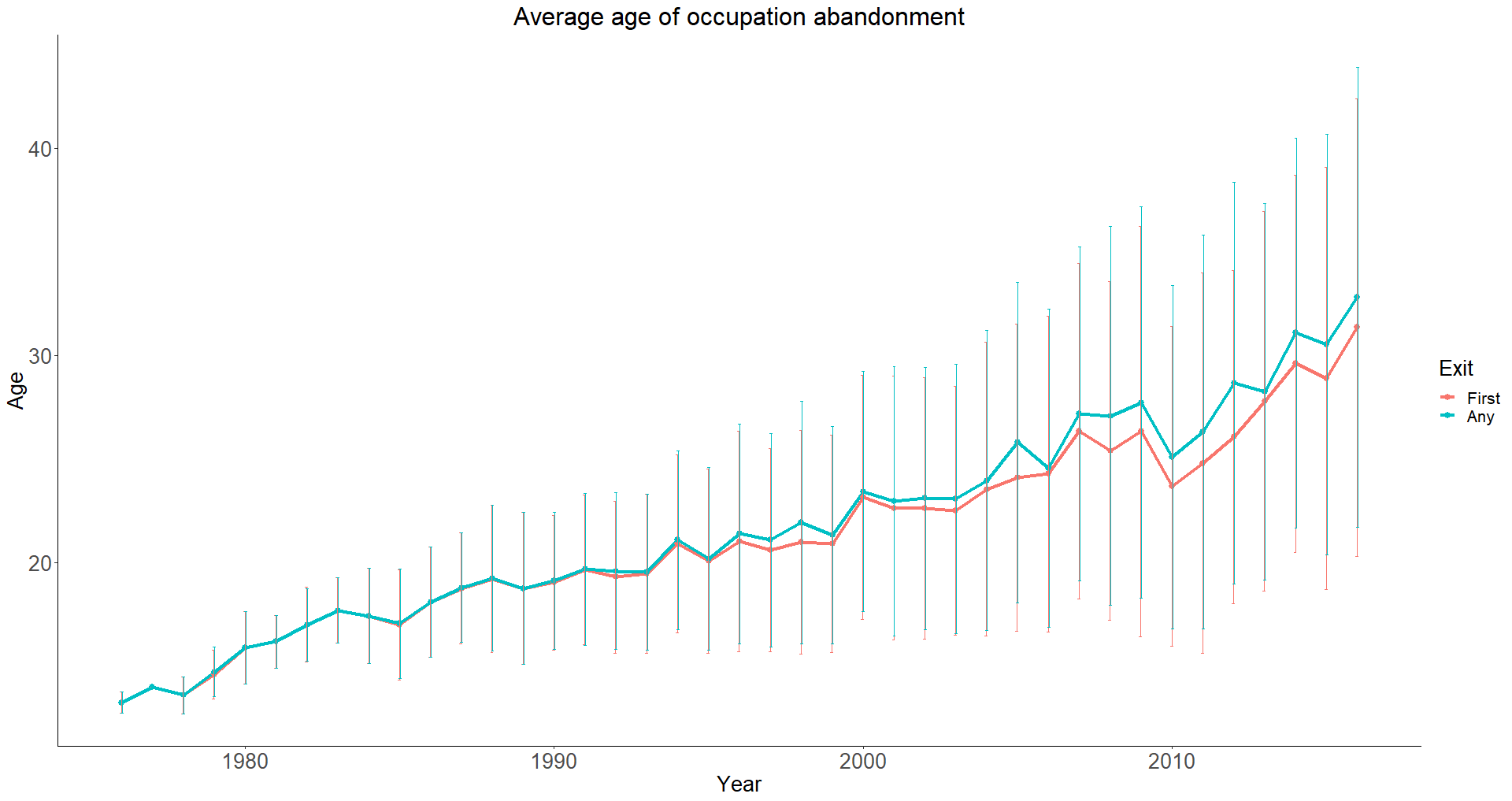

Supplement: S1 Fig — We also observed that the average age of departure from agrarian occupations shows a consistent increment. For example, between 1980 and 1990, it ranged between 15 and 19 years, whereas from 2000 to 2017, it fluctuated between 23 and 35 years. This trend suggests a gradual abandonment of agrarian occupations, where younger individuals left first and are recently followed by older individuals (S1 Fig). (TIF) [file pone.0313891.s001.tif]
